# Anomalous Stranski-Krastanov growth of (111)-oriented quantum dots with tunable wetting layer thickness

**DOI:** 10.1038/s41598-019-54668-z

**Published:** 2019-12-03

**Authors:** Christopher F. Schuck, Simon K. Roy, Trent Garrett, Qing Yuan, Ying Wang, Carlos I. Cabrera, Kevin A. Grossklaus, Thomas E. Vandervelde, Baolai Liang, Paul J. Simmonds

**Affiliations:** 1 Micron School of Materials Science & Engineering, Boise State University, Boise, Idaho 83725 USA; 2 Department of Physics, Boise State University, Boise, Idaho 83725 USA; 3 College of Physics Science & Technology, Hebei University, Baoding, 071002 P.R. China; 4 Center for Research in Sciences, Research Institute in Basic and Applied Sciences, Autonomous University of the State of Morelos, Av. Universidad 1001, 62209 Cuernavaca, Morelos Mexico; 5 Department of Electrical and Computer Engineering, Tufts University, 161 College Avenue, Medford, Massachusetts 02155 USA; 6 Present Address: Materials Growth Facility, University of Delaware, Newark, Delaware 19716 USA

**Keywords:** Nanoscale materials, Quantum physics, Semiconductors, Surfaces, interfaces and thin films, Electronics, photonics and device physics

## Abstract

Driven by tensile strain, GaAs quantum dots (QDs) self-assemble on In_0.52_Al_0.48_As(111)A surfaces lattice-matched to InP substrates. In this study, we show that the tensile-strained self-assembly process for these GaAs(111)A QDs unexpectedly deviates from the well-known Stranski-Krastanov (SK) growth mode. Traditionally, QDs formed via the SK growth mode form on top of a flat wetting layer (WL) whose thickness is fixed. The inability to tune WL thickness has inhibited researchers’ attempts to fully control QD-WL interactions in these hybrid 0D-2D quantum systems. In contrast, using microscopy, spectroscopy, and computational modeling, we demonstrate that for GaAs(111)A QDs, we can continually increase WL thickness with increasing GaAs deposition, even after the tensile-strained QDs (TSQDs) have begun to form. This anomalous SK behavior enables simultaneous tuning of both TSQD size and WL thickness. No such departure from the canonical SK growth regime has been reported previously. As such, we can now modify QD-WL interactions, with future benefits that include more precise control of TSQD band structure for infrared optoelectronics and quantum optics applications.

## Introduction

Historically, researchers have classified epitaxial growth into three modes: two dimensional (2D) layer-by-layer growth (Frank-van der Merwe), 3D island formation (Volmer-Weber), or layer-plus-island growth (Stranski-Krastanov (SK))^1,2^. We can take advantage of SK growth to achieve strain-driven self-assembly of dislocation-free semiconductor quantum dots (QDs) with tunable optoelectronic properties^2–6^. SK QD growth proceeds as follows: (i) a 2D wetting layer (WL) forms; (ii) at some critical thickness, *t*_*c*_, 3D QDs nucleate and self-assemble on the WL; and (iii) the QDs grow while the WL thickness is pinned at *t*_*c*_ ^2,7^.

The WL quantum well (QW) behaves essentially as a carrier reservoir, interconnecting all QDs in a layer. WL thickness can thus have significant influence on QD band structure, affecting emission wavelength^8–11^, band edge profile^11^, carrier confinement depth^8,9^, excited state and charged exciton energy levels^8,9^, QD-WL interaction strength^8^, and WL interface fluctuations^12,13^. Although these effects have important implications for QD devices^8–12,14,15^, our ability to take advantage of them is hindered by the fact that the maximum WL thickness, *t*_*c*_, is a fixed parameter in conventional SK self-assembly^2,7–11,15^. For example, for compressively strained InAs on GaAs, once the InAs WL thickness reaches *t*_*c*_ ~ 1.6 monolayers (ML), all additional InAs deposited contributes to QD self-assembly^2,16,17^.

To sidestep this constraint, researchers have developed some creative approaches to manipulate WL thickness, including high-temperature WL desorption^17^, modified droplet epitaxy^9^, and unstrained inverted QD structures^8,18^. However, the ability to simply tune WL thickness in a single SK growth step without additional processing, would provide a scalable route to optoelectronic devices with complete control over QD-WL interactions.

In this paper we demonstrate that an anomalous SK growth mode governs the self-assembly of GaAs tensile-strained QDs (TSQDs), wherein the WL thickness is tunable. We grow GaAs TSQDs on In_0.52_Al_0.48_As(111)A (hereafter InAlAs) by molecular beam epitaxy (MBE). The GaAs TSQDs exhibit unique properties that derive from the tensile strain, as well as their (111) orientation^19–22^. Growth proceeds via the initial formation of a 2D WL, followed by a transition to 3D TSQD self-assembly. However, using a combination of microscopy, spectroscopy and computational modeling, we demonstrate that GaAs deposition beyond *t*_*c*_ increases both QD size *and* WL thickness, in contrast with conventional SK growth.

## Results

### Atomic force microscopy (AFM): WL Growth Beyond *t*_*c*_

**Figure 1:**
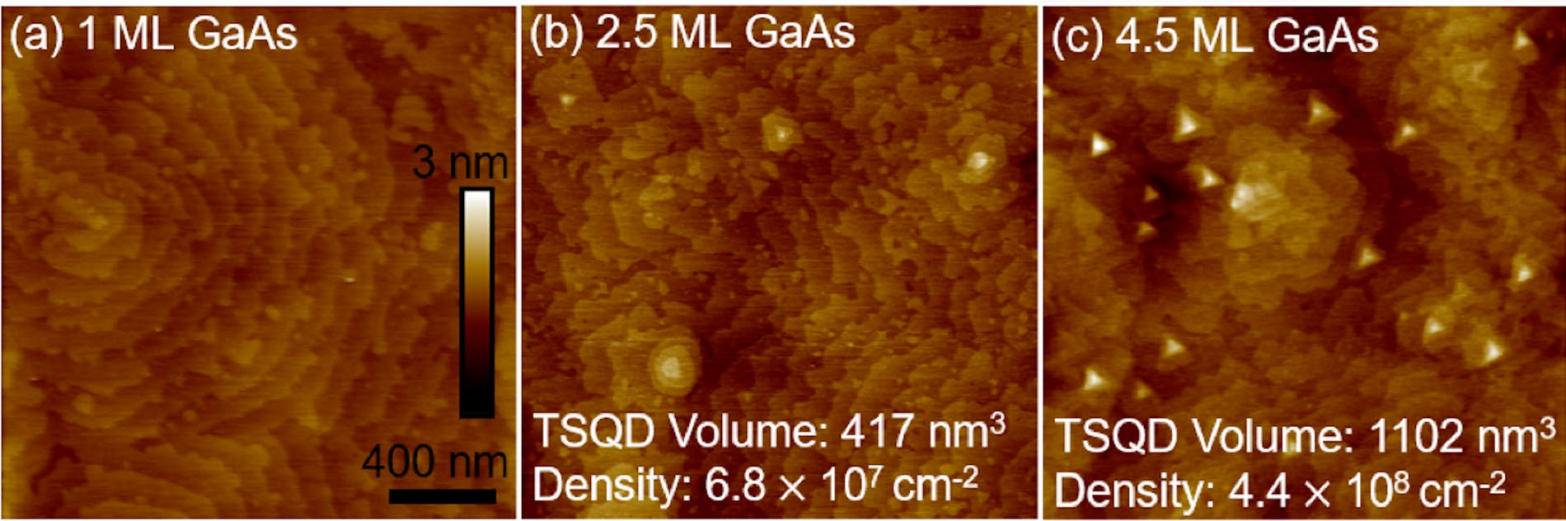
2 × 2 µm^2^ AFM images showing evolution of the surface morphology with increasing GaAs deposition amount: (**a**) 1 ML, (**b**) 2.5 ML, and (**c**) 4.5 ML.

We grew a series of GaAs/InAlAs(111)A samples for which we varied the GaAs deposition amount from 0–4.5 ML (see Methods section for details of sample growth and characterization). Using AFM, we see that for deposition <2.5 ML, GaAs forms a 2D WL with ML-high terraces and no TSQDs [Fig. 1(a)]. These highly terraced surfaces are typical of (111)A-oriented materials^23^. At *t*_*c*_ = 2.5 ML GaAs, a low density of small 3D tetrahedral TSQDs appear [Fig. 1(b)]. This 2D-to-3D transition is consistent with SK growth. The fact that the critical thickness is larger than the well-known value of ~1.6 ML for InAs/GaAs(001) QDs^16^ is mainly a function of the lower strain in the GaAs/InAlAs(111)A TSQD system. InAs on GaAs experiences 7.2% compressive strain, while GaAs on InAlAs experiences 3.8% tensile strain. With less strain available, a thicker, 2.5 ML wetting layer is required to drive the SK transition from 2D to 3D growth. By 4.5 ML GaAs, TSQD volume and areal density increase, but remain low compared to traditional QD materials systems [Fig. 1(c)]^2,24^. Figure 1(c) shows that for larger GaAs deposition amounts, TSQD size can become bimodally distributed, a fact that we have reported previously for GaAs/InAlAs(111)A TSQDs^19^. In this respect, TSQDs behave similarly to traditional self-assembled QD systems such as InAs/GaAs(001)^25^. The triangular shape of the GaAs TSQDs [Fig. 1(c)] mirrors the threefold symmetry of the underlying InAlAs(111)A surface. The sides of the triangular GaAs(111)A TSQDs are perpendicular to the $[\bar{1}\bar{1}2]$, $[\bar{1}2\bar{1}]$, and $[2\bar{1}\bar{1}]$ directions: the so-called “A” steps, with two dangling bonds per edge atom^26^.

For each sample in this study, Table 1 shows the WL thickness and, for ≥2.5 ML deposition, the average TSQD height, diameter and areal density, as measured by AFM. To calculate WL thickness from the amount of GaAs deposited, we first use the fact that 1 ML of GaAs(111) pseudomorphically strained to In_0.52_Al_0.48_As(111) has an interplanar spacing of 0.323 nm (see also the section on electron microscopy below). We then subtract the volume of GaAs contained in any TSQDs to find the total WL thickness (see Methods and Supplementary Information). The critical thing to note is that for all samples, the total volume of GaAs within the TSQDs is ≤1% of the total amount of GaAs deposited. It seems that most GaAs must therefore join the WL, even for deposition exceeding *t*_*c*_ = 2.5 ML.

**Table 1:** WL thicknesses, and average TSQD sizes and areal densities for each sample.

GaAs deposition (ML)	WL thickness (nm)	TSQD height (nm)	TSQD diameter (nm)	TSQD areal density (µm^−2^)
0.5	0.162	—	—	—
1.0	0.323	—	—	—
1.5	0.485	—	—	—
2.5	0.808	0.472 ± 0.126	68.6 ± 10.3	0.68
3.0	0.969	0.591 ± 0.117	58.6 ± 9.0	1.00
3.5	1.131	0.757 ± 0.182	60.5 ± 10.1	1.88
4.0	1.279	1.339 ± 0.253	59.2 ± 8.9	10.76
4.5	1.447	1.386 ± 0.219	62.7 ± 10.6	4.40

### Photoluminescence (PL) spectroscopy: Evidence for unusual WL behavior

**Figure 2:**
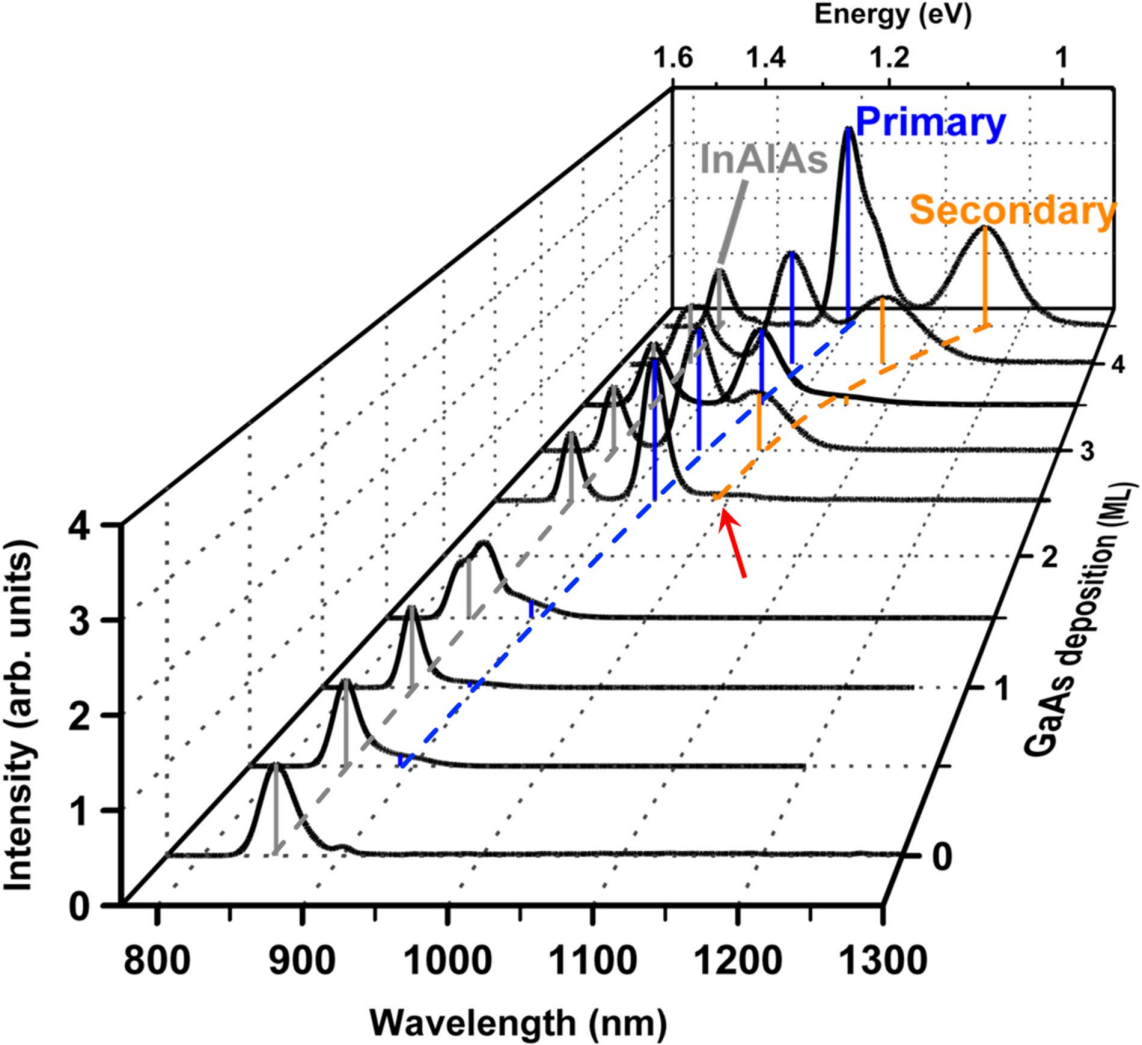
PL emission at 7 K from TSQD samples, showing spectral evolution with increasing GaAs deposition amount. PL excitation density is 9.5 W/cm^2^. The red arrow indicates the weak onset of secondary peak PL emission for the 2.5 ML GaAs sample.

The GaAs/InAlAs(111)A TSQD samples exhibit three spectral features [Fig. 2]. The 0 ML GaAs reference sample has an InAlAs emission peak at 852 nm. The small peak at 900 nm arises from shallow donor-shallow acceptor recombination in the InP substrate^27,28^. For 0.5 ML GaAs deposition, a shoulder emerges on the long wavelength side of the InAlAs peak and resolves into a discrete peak at 2.5 ML, which we refer to as the ‘primary’ peak. As we raise the amount of GaAs from 0.5–4.5 ML, the primary peak wavelength increases from 890–1000 nm. At 2.5 ML GaAs deposition, a third peak develops at yet longer wavelength, which we refer to as the ‘secondary’ peak (see red arrow in Fig. 2). The emergence of this broader, secondary peak coincides with the appearance of TSQDs [Fig. 1(b)]. Raising the amount of GaAs from 2.5–4.5 ML, increases the secondary peak wavelength from 987–1160 nm.

Due to the 3.8% tensile strain, both the primary and secondary GaAs-related PL peaks are longer in wavelength than band-to-band emission from bulk GaAs (816 nm at 7 K)^20,28,29^. Guided by PL studies of conventional, compressively strained InAs QDs, we tentatively attribute the primary peak to the GaAs WL, and the secondary peak to the GaAs TSQDs^30^. GaAs TSQDs have typical areal densities in the range 1–10 µm^–2^ (see Table 1 and ref. ^19^). Given that the spot size of our PL excitation laser is ~78.5 µm^2^, we would therefore expect to excite anywhere from 80–800 TSQDs, depending on the particular sample. The low intensity of the resulting secondary TSQD peak, compared to the primary WL peak, is the result of the low areal density of GaAs TSQDs. Traditional In(Ga)As/GaAs QDs have typical areal densities of 100 µm^–2^ and PL from the WL is often weak compared with QD emission, or not observed at all^31^.

The secondary peak redshifts as we deposit more GaAs, consistent with increasing TSQD volume producing quantum size effects. However, the primary peak also redshifts after TSQDs appear, which is completely unexpected. If the primary peak corresponds to WL emission as we conjecture, this wavelength increase supports the indications from AFM that the WL QW continues to grow thicker beyond *t*_*c*_. Such behavior is inconsistent with standard SK growth: emission wavelength is fixed for a WL whose thickness is pinned at *t*_*c*_ ^31^.

**Figure 3:**
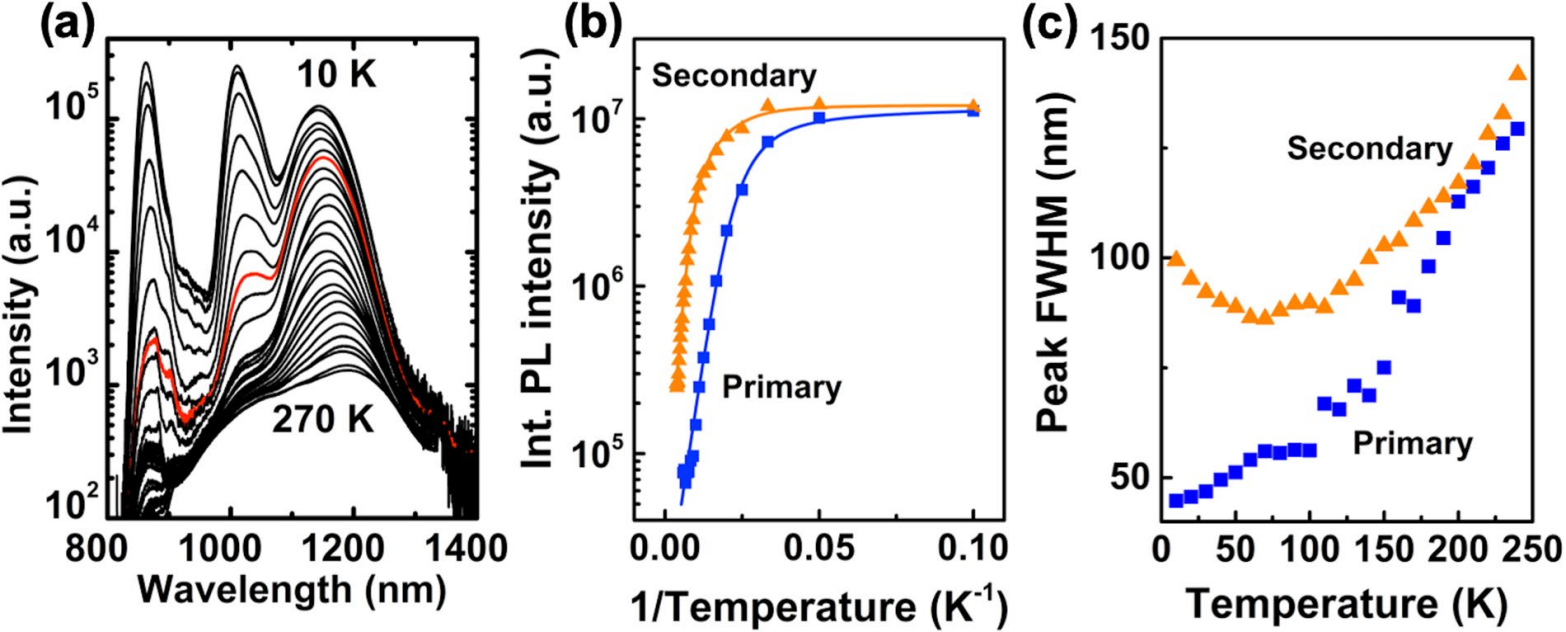
(**a**) Temperature-dependent PL from 4.5 ML GaAs TSQD sample. The red spectrum was collected at 80 K. (**b**) Integrated intensities of primary and secondary PL peaks plotted against inverse temperature. Solid lines are fits from Eq. 1. (**c**) FWHM of primary and secondary peaks in (**a**) as a function of temperature. Excitation density is 9.5 W/cm^2^.

Given this unexpected result, we must exclude alternative causes of the primary PL peak. Possible origins include TSQD excited-state emission^32^, emission from phase-separated In-rich nanoclusters in the InAlAs^33^, or emission from a family of smaller GaAs TSQDs due to a bimodal size distribution^19^. The primary peak persists even for PL excitation densities as low as 0.3 W/cm^2^, which precludes excited state emission as the cause [see Supplementary Fig. S1]. We rule out the other two alternative explanations for the primary peak using temperature-dependent PL [Fig. 3(a)]. After Gaussian fitting, we plot integrated primary and secondary peak intensities, *I*, against inverse temperature, 1/*T* [Fig. 3(b)]. For each curve we extract two activation energies, *E*_1_ and *E*_2_, using a biexponential model^33^:

1 $$I(T)={I}_{0}/(1+{C}_{1}{e}^{-{E}_{1}/{k}_{B}T}+{C}_{2}{e}^{-{E}_{2}/{k}_{B}T})$$

where *I*_0_ is the integrated intensity at *T* = 0 K, *C*_1,2_ are constants, and *k*_*B*_ is the Boltzmann constant. The resulting fits agree well with the experimental data [Fig. 3(b)]. For the secondary peak, we obtain *E*_1_ = 46 meV and *E*_2_ = 13 meV. For the primary peak, values of *E*_1_ = 24 meV and *E*_2_ = 9 meV rule out In-rich InAlAs nanoclusters as the cause, since we have previously measured activation energies of *E*_1_ = 34 meV and *E*_2_ = 5 meV for these QD-like features^33^.

As we raise the temperature from 10–80 K, the full width at half maximum (FWHM) of the secondary peak decreases from 100–85 nm, before increasing again at higher temperatures [Fig. 3(c)]. PL from smaller TSQDs is quenched as weakly confined carriers thermalize into larger dots with lower energy ground states. This “u-shaped” temperature dependence of the FWHM is a characteristic feature of QD arrays, and confirms that TSQD emission produces the secondary peak^28,34^.

The fact that the primary peak is still pronounced at 80 K [red curve in Fig. 3(a)], means it cannot be due to emission from a discrete population of smaller TSQDs, since they would be depleted of carriers by this temperature. In contrast with the secondary peak, the FWHM of the primary peak increases continuously with measurement temperature [Fig. 3(c)]. This monotonic increase in FWHM is consistent with thermal broadening of QW emission due to increased electron-phonon scattering, and confirms that this peak is not related to TSQD emission.

Having confirmed that the primary and secondary peaks originate in the WL and TSQDs respectively, we return to the unusual result that WL growth continues after the SK transition to TSQD formation.

### STEM/EELS: anomalous SK growth confirmed

Analysis of the 2.5 and 4.5 ML samples with a combination of scanning transmission electron microscopy (STEM) and electron energy loss spectroscopy (EELS) confirms a modified SK growth mode. Directly resolving the WLs and TSQDs by STEM is difficult due to the low Z-contrast between GaAs and the InAlAs matrix. Imaging the WL in the 2.5 ML GaAs sample is additionally challenging due to its narrow width. However, we can observe the 4.5 ML GaAs TSQDs directly with bright-field TEM due to increased strain contrast (Supplementary Fig. S2). The TSQDs are dislocation-free despite the high tensile strain.

**Figure 4:**
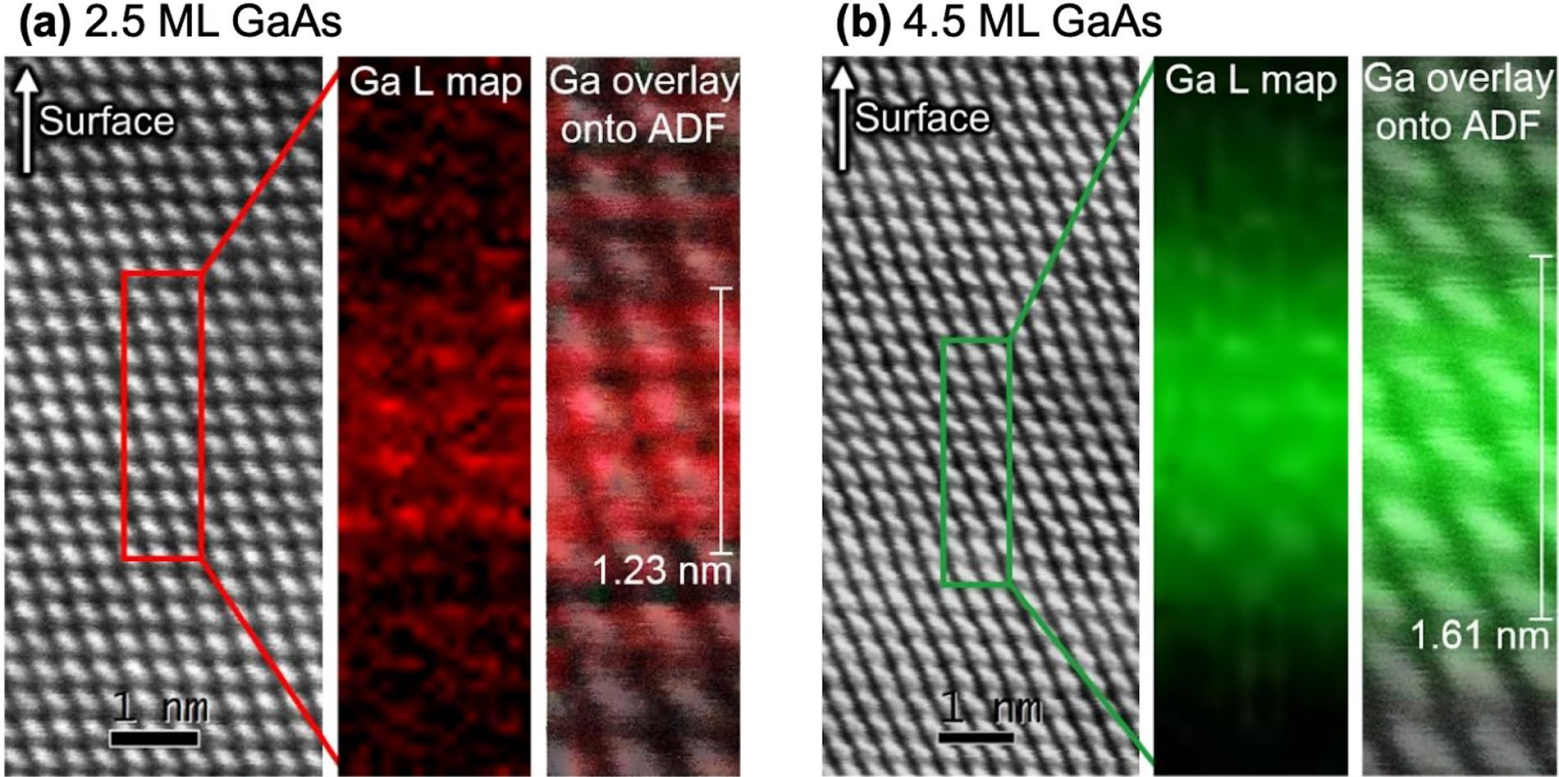
STEM ADF images of (**a**) 2.5 ML and (**b**) 4.5 ML GaAs TSQD samples, indicating the areas used for EELS compositional mapping of the Ga L signal (central panels). Right-hand panels show EELS maps overlaid on corresponding ADF images.

We used STEM/EELS mapping of the Ga L edge signal to identify the position of the GaAs WL in both the 2.5 ML and 4.5 ML samples. To quantitatively analyze the thickness of these WLs, for each sample we took multiple intensity traces across the EELS maps. For each trace, we found the FWHM of the elevated Ga L signal corresponding to the WL. After calculating the average and standard deviation of the multiple FWHM measurements, we corrected those values to account for sample drift during mapping, which is visible in subsequent annular dark-field (ADF) STEM images from the area marked by carbon deposition due to the electron beam. We took the final drift-corrected average FWHM value to be the WL thickness. Applying this analysis method to the Ga L edge signal map for the 2.5 ML sample [Fig. 4(a)] gives an average WL thickness of 1.23 ± 0.11 nm. If we assume that the GaAs WL is pseudomorphically strained in-plane to the surrounding In_0.52_Al_0.48_As, then the 0.326 nm unstrained (111) interplanar spacing of GaAs will be compressed to approximately 0.323 nm. This compressed (111) GaAs interplanar spacing is equivalent to 1 ML for this orientation. Therefore, the WL thickness we determined for the 2.5 ML sample corresponds to 3.81 ± 0.34 ML GaAs fully strained in-plane to the InAlAs matrix. Overlaying the ADF image with the EELS Ga L edge map in Fig. 4(a) allows us to visually confirm that the GaAs WL in the 2.5 ML sample is 3–4 ML thick, in agreement with our analysis. Repeating this process for the Ga L edge signal map of the 4.5 ML sample [Fig. 4(b)] gives a drift-corrected average thickness of 1.61 ± 0.08 nm, which corresponds to 4.98 ± 0.24 ML GaAs strained in-plane to the InAlAs matrix. Overlaying the ADF image with the EELS Ga L edge map in Fig. 4(b) again confirms that the GaAs WL is 4–5 ML thick, in agreement with our analysis.

The fact that these measured WL thicknesses are greater than the nominal GaAs deposition amounts is likely due to a combination of low Ga L signal, monolayer-scale thickness variation through the TEM sample, and out-diffusion of Ga into the surrounding InAlAs matrix. It is reasonable to assume a certain amount of interdiffusion between the Ga in our TSQDs and the In in the InAlAs matrix. Al does not participate; previous studies of interdiffusion at InGaAs/InAlAs interfaces show that in terms of atomic mobility, In > Ga > Al, i.e., in inverse proportion to their lattice bond strengths^35^. We therefore expect some Ga-In interdiffusion driven by the concentration gradient, just as we see in traditional InAs/GaAs(001) QDs^36^. However, putting these uncertainties to one side, the STEM/EELS measurements in Fig. 4 show conclusively that WL thickness increases beyond *t*_*c*_ from 2.5 ML to 4.5 ML GaAs (deposited thicknesses), consistent with our AFM and PL results.

### TSQD and WL computational model: agreement with experiment

**Figure 5:**
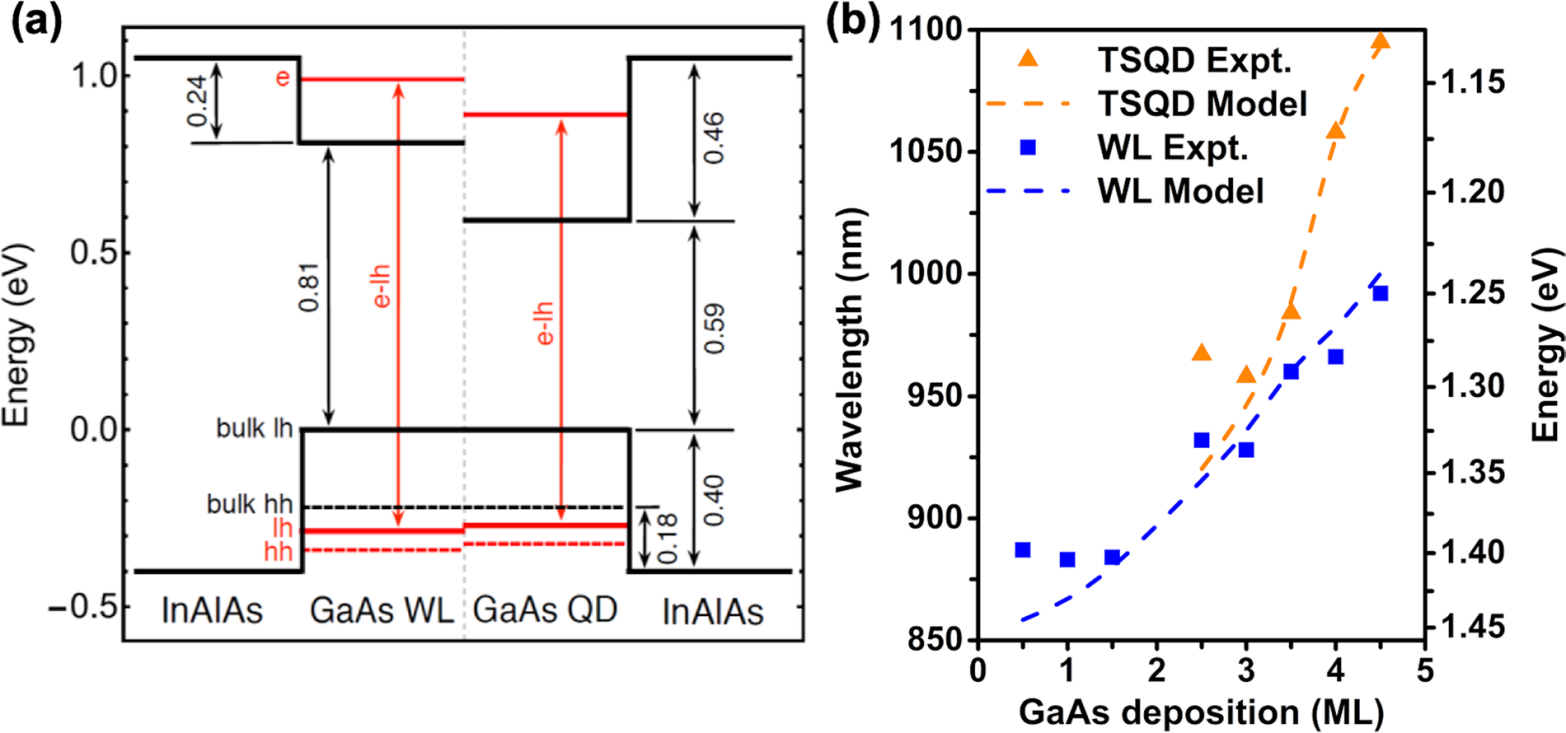
(**a**) Calculated band diagram for a 4 ML GaAs(111) TSQD under 3.8% biaxial tensile strain, using experimental values for height and diameter of 1.339 nm and 59.2 nm, respectively, together with a GaAs WL QW that is 1.279 nm thick (see Table 1). Ground state e-lh emission is in red. (**b**) Comparison between experimental and calculated peak PL emission as a function of increasing GaAs deposition amount, for TSQDs and WL QWs. PL excitation density is 3000 W/cm^2^.

Band structure modeling confirms our experimental conclusions of anomalous SK growth. Tensile strain and quantum confinement act in opposition, respectively reducing and increasing the effective TSQD band gap^20,28,37^. Given the “push-pull” nature of these contributions, TSQD band structure as a function of size is fairly complex. To compute the strain-induced modification of the GaAs band gap in these tetrahedral TSQDs (Supplementary Fig. S3(a)), we resolve the total strain into its hydrostatic and biaxial components. The hydrostatic component acts on the band edges, thereby changing the band gap. The biaxial component acts on the valence bands. Decoupling the conduction and valence bands, the strain-induced changes to the band edges at the Γ point are given by^38^,

2a $$\delta {E}_{c}={a}_{c}(2{\varepsilon }_{\parallel }+{\varepsilon }_{\perp }),$$

2b $$\delta {E}_{v}={a}_{v}(2{\varepsilon }_{\parallel }+{\varepsilon }_{\perp })\pm b({\varepsilon }_{\parallel }-{\varepsilon }_{\perp }),$$

where + (−) applies to the light hole, *lh* (heavy hole, *hh*) band. *a*_*c*_ and *a*_*v*_ are the conduction and valence band hydrostatic deformation potentials. *b* is the shear deformation potential. The in-plane, *ε*_||_, and perpendicular, *ε*_⊥_, strains, are related by the Poisson ratio for the (111) orientation^39^. Tensile strain reduces the GaAs band gap and breaks the valence band degeneracy at the Γ point, raising the *lh* band above the *hh* band [Fig. 5(a)].

TSQD-WL interactions are clearly more complex in this anomalous SK system where the WL thickness changes, compared with SK-grown QDs for which the WL is fixed at *t*_*c*_. We therefore adopt an approach consistent with previous studies that have considered confinement in QD-WL systems as a whole. We assume an effective TSQD height of *h*_*QD*_ = *h*_*AFM*_ + *h*_*WL*_ (illustrated in Supplementary Fig. S3(b–d)), where *h*_*AFM*_ and *h*_*WL*_ are taken from Table 1. Wang *et al*. investigated QDs formed in back-filled nanoholes, and found that a thinner WL (and hence smaller *h*_*QD*_) led to a systematic blue-shift in QD emission, even though QD shape and lateral size was fixed^8^. The authors attributed this behavior to “cross talk” between vertical confinement potential and lateral confinement potential. For QDs formed from monolayer high fluctuations in a QW thickness, there is similar leakage of the confined wave functions both in-plane beyond the physical dimensions of the dots, and also out-of-plane into the barriers^40^.

We calculate WL and tetrahedral TSQD ground state energy levels for each sample (for more details on our model see the Methods section). Figure 5(a) shows an example band structure calculated for a 4 ML GaAs TSQD, for which we can see the quantum confined states for both the 0D QD, and the 2D WL (QW). Our model parameters for GaAs(111) are *b* = −2 eV, and *a*_*v*_ = −2.1^41^. For the WL QW we use *a*_*c*_ = −7.4 eV^42^. For the TSQDs, our model assumes a constant average strained potential throughout a TSQD and does not account for band mixing. Because of these simplifications, we use *a*_*c*_ as a fitting parameter to couple strain to electronic structure. For the TSQDs, *a*_*c*_ = −11.5 eV gave the closest fit to our PL data^42^. Calculated WL and TSQD transition energies agree closely with experimental PL data as a function of GaAs deposition [Fig. 5(b)]. Our model captures the redshift of both the TSQD *and* WL peaks as they continuously grow thicker, even beyond *t*_*c*_ = 2.5 ML.

We suspect the small divergence for the WL at 0.5 ML and 1 ML between the model and the experimental data comes from small uncertainties in our estimates of the total amount of GaAs deposited, and hence the WL thicknesses. These errors will be magnified for very low deposition amounts. Our model also ignores any Ga-In interdiffusion between the GaAs WL and InAlAs barrier, which would modify both the WL thickness and composition. We attribute the divergence between the model and the experiment for the 2.5 ML TSQDs to errors in our AFM measurements of TSQD size. These errors are amplified for these very small TSQDs with low areal density. Moreover, capping with InAlAs may modify both the shape and size of the TSQDs as the result of Ga-In interdiffusion, which could have more of an impact for these tiny 2.5 ML TSQDs. Our theoretical and experimental data converge at higher coverage as these sources of error are minimized. Overall, the transition energies predicted by our model agree well with experiment for both WL and TSQDs, and confirm SK-like growth with unusual wetting layer behavior.

## Discussion: An Anomalous SK Growth Mode

Although previous studies have hinted at potential anomalies in the SK-based self-assembly of TSQDs^19,20^, the results presented here give a concrete picture. The tensile-strained self-assembly of GaAs(111)A TSQDs allows us to combine highly controllable SK growth with the additional benefit of a WL whose thickness is tunable. The growth mode we observe is perhaps best described as a hybrid of the SK_1_ and SK_2_ growth modes outlined by Barabási^7^. Adatoms deposited after *t*_*c*_ has been reached bind preferentially to the growing WL, suggesting TSQD formation provides only a subtle reduction in the free energy^7^. This is consistent with the small volume and low areal density of TSQDs, compared to QDs formed during conventional SK growth^2,19,24^.

In an effort to provide a mechanism for this anomalous SK growth, it is helpful to consider how intermixing dictates the critical thickness of the wetting layer during compressively strained QD self-assembly^43,44^. In the traditional InGaAs/GaAs(001) QD system, In-Ga intermixing at the interface dilutes the indium composition of the deposited layer below its nominal value. However, as the largest atomic species, indium also undergoes vertical segregation driven by the strain, resulting in indium enrichment in the surface monolayer^43^. As growth proceeds, the indium content in this surface layer gradually reaches some critical concentration of 80–85%^43^, at which point the strain is large enough for the SK transition from 2D to 3D growth to occur^45,46^. The lower the nominal indium composition in the deposited InGaAs, the thicker the wetting layer needs to be before this critical surface concentration is achieved.

We can apply this same idea to the growth of GaAs/InAlAs(111)A TSQDs. At the onset of growth, Ga-In intermixing occurs between the deposited GaAs and the underlying InAlAs. Indeed, this process may be enhanced by the fact that the InAlAs surface is likely to be indium-rich due to surface segregation^47,48^. The result is that close to the interface, the emerging wetting layer will in fact be Ga(In)As, while the top few monolayers of the underlying surface will be InAl(Ga)As. Diluting the as-deposited GaAs with indium lowers the Ga concentration at the wetting layer surface below the threshold for SK growth. Furthermore, this intermixing lowers the lattice mismatch and hence the tensile strain, stabilizing the planar morphology of the wetting layer as it begins to grow^44^.

As GaAs deposition continues, the interface becomes buried, and the Ga concentration at the surface of the growing Ga(In)As wetting layer gradually increases. However, this process is likely to be retarded due to the continuing surface segregation of indium in the uppermost monolayer of the Ga(In)As wetting layer^43^. For this reason, it is possible that the Ga concentration one monolayer below the wetting layer surface may actually be slightly higher than at the surface itself. Finally, when ~2.5 ML of GaAs has been deposited, a critical Ga composition is reached with sufficient tensile strain to trigger the SK 3D-islanding transition, and TSQDs begin to form.

It is the indium surface segregation during wetting layer growth that could help explain the anomalous SK growth mode we observe for GaAs/InAlAs TSQDs. Even if the Ga concentration in the wetting layer subsurface has crossed the threshold for TSQD self-assembly, the presence of excess indium in the surface monolayer could “pin” the composition slightly below the critical concentration. As a result, planar wetting layer growth could continue at the surface, even as 3D TSQDs from the comparatively Ga-rich subsurface get larger.

## Conclusions

In summary, after investigating the structural and optical properties of GaAs/InAlAs(111)A TSQDs, we conclude that self-assembly occurs via a modified version of the well-known SK mechanism. In this anomalous SK growth mode, the WL continues to increase in thickness, even after the TSQDs have begun to grow. Now that we can grow TSQDs on top of wetting layers of different thickness, our next goal is to find ways to decouple WL thickness and TSQD size. Controlling each separately will allow us to tune TSQD and WL states on or off resonance. To this end, future avenues we will explore include changing the InAlAs barrier composition to modify both the tensile strain and the amount of indium available for interdiffusion, adjusting the MBE growth conditions partway through TSQD self-assembly, or post-growth annealing, which we have previously shown can reduce a TSQD’s height while increasing its diameter^19^. The ability to control WL thickness and QD size independently will provide a new avenue for future studies into WL-QD interactions. We anticipate the development of optoelectronic devices that could take advantage of this high degree of structural tunability.

## Methods

### Sample growth

We grew all samples for this study by MBE, on Fe-doped (i.e., semi-insulating), nominally on-axis InP(111)A substrates for consistency with our previous studies of this material system^19,20,23^. Samples consist of a lattice-matched In_0.52_Al_0.48_As bottom barrier, followed by 0.5–4.5 ML embedded GaAs TSQDs for optical analysis, then an In_0.52_Al_0.48_As capping layer, and finally 0.5–4.5 ML surface GaAs TSQDs for structural analysis. TSQD growth conditions are optimized for high intensity photon emission^19–21,49^. See Supplementary Information for MBE growth conditions and sample structure details (Supplementary Fig. S4).

### Sample characterization

We measured the sizes of ≥100 tetrahedral surface TSQDs per sample with AFM. Subtracting the total volume of the TSQDs from the total amount of GaAs deposited gives the WL volume per unit area, and hence WL thickness. To explore the optical characteristics of the buried TSQDs, we use PL spectroscopy (excitation laser wavelength = 532 nm, excitation density = 0.3–3000 W/cm^2^, temperature = 7–300 K) using a 20× objective lens to give a laser spot with a calibrated FWHM of ~10 µm, and so an area of ~78.5 µm^2^. We compare PL from these coupled WL-QD systems, to a bulk InAlAs(111)A (0 ML GaAs) reference sample^19^.

To image WL and TSQD structure we use high-resolution cross-sectional transmission electron microscopy (XTEM) in bright-field conditions (diffraction contrast), and STEM using bright-field and ADF imaging modes. To measure WL thickness, we use simultaneous STEM ADF imaging and high spatial resolution EELS composition mapping using the Ga L and In M edges.

### Computational modeling

We deduce carrier confinement potentials, *U*, by adding the strain-induced changes in Eqs. (2a, 2b) to the GaAs/InAlAs heterostructure band offsets. We take the offset ratio for the unstrained bands from the difference in absolute energetic position of the average valence band^39^.

We treat the WL as a QW and use the envelope function approximation to compute the conduction band energy levels. We describe electron behavior in the QW using the Schrödinger equation within the effective mass approximation. To determine the QW energy levels in the *lh* and *hh* bands under tensile strain, we use a 4 × 4 ***k·p*** Kohn-Luttinger Hamiltonian^38^. For each sample, we use a value for the WL thickness calculated from the GaAs deposition amount (see Table 1).

To establish TSQD electronic structure, we adopt a single-band, constant-confining-potential model, using the Hamiltonian operator to calculate electron and hole energy levels:

3 $$H({\bf{r}})=-\,\frac{{\hslash }^{2}}{2}({\rm{\nabla }}\frac{1}{{m}^{\ast }({\bf{r}})}{\rm{\nabla }})+U({\bf{r}})$$

To mirror their physical shape [Fig. 2(c)], we model each TSQD as a tetrahedron whose base is an equilateral triangle [Supplementary Fig. S3(a)]. The dimensions of these tetrahedra come from AFM measurements for each sample (see Table 1), with typical height-to-width ratios of 0.01–0.02. We expand the envelope function *ψ*(***r***), as a linear combination of a basis set, {*ϕ*}, of the solutions of the cuboidal QD, *L*_*x*_*L*_*y*_*L*_*z*_, with infinite barrier height, $\psi ({\boldsymbol{r}})=\sum _{lmn}{a}_{lmn}{\varphi }_{lmn}({\boldsymbol{r}})$ [Supplementary Fig. S3(a)]. Following Van de Walle’s approach^39^, we obtain the TSQD energy levels by solving the matrix equation

$$({M}_{lmnl^{\prime} m^{\prime} n^{\prime} }-E{\delta }_{ll^{\prime} }{\delta }_{mm^{\prime} }{\delta }_{nn^{\prime} }){a}_{lmn}=0,$$

where we have used wave function orthonormality. The matrix elements ${M}_{lmnl^{\prime} m^{\prime} n^{\prime} }$ are given by^50^:

4 $$\begin{array}{ccc}{M}_{lmnl^{\prime} m^{\prime} n^{\prime} } & = & \int {\psi }_{l^{\prime} m^{\prime} n^{\prime} }^{\ast }\,H({\bf{r}})\,{\psi }_{lmn}\,\text{d}{\bf{r}}\\  & = & [\frac{{\hslash }^{2}{\pi }^{2}}{2{m}_{b}^{\ast }}(\frac{ll^{\prime} }{{L}_{x}^{2}}+\frac{mm^{\prime} }{{L}_{y}^{2}}+\frac{nn^{\prime} }{{L}_{z}^{2}})+U]{\delta }_{ll^{\prime} }{\delta }_{mm^{\prime} }{\delta }_{nn^{\prime} }\\  &  & +\,\frac{{\hslash }^{2}}{2}(\frac{1}{{m}_{d}^{\ast }}-\frac{1}{{m}_{b}^{\ast }}){\int }_{D}{\rm{\nabla }}{\varphi }_{l^{\prime} m^{\prime} n^{\prime} }^{\ast }{\rm{\nabla }}{\varphi }_{lmn}\,\text{d}{\bf{r}}-U{\int }_{D}{\varphi }_{l^{\prime} m^{\prime} n^{\prime} }^{\ast }{\varphi }_{lmn}\,\text{d}{\bf{r}}\end{array}$$

where integration is over the TSQD region (*D)*. ${m}_{d}^{\ast }$ and ${m}_{b}^{\ast }$ are the carrier effective masses in the GaAs TSQDs and InAlAs barriers respectively. To ensure energy eigenvalues are independent of the outer box size, we use values *L*_*x*_ = *L*_*y*_ = 150 nm and *L*_*z*_ = *L*_*x*_/2. To ensure convergence within 1 meV for all TSQD sizes, we use a basis set of 19^3^ wavefunctions^51^. We then solve the matrix ${M}_{lmnl^{\prime} m^{\prime} n^{\prime} }$ numerically using the Monte Carlo method.

## Supplementary information

Suplementary Information

## Data Availability

The datasets used and/or analyzed during the current study are available from the corresponding author on reasonable request.
