# Supplementary material for: Anomalous Stranski-Krastanov growth of (111)-oriented quantum dots with tunable wetting layer thickness
Source: Sci Rep. 2019 Dec 3;9:18179. doi: 10.1038/s41598-019-54668-z (PMC6890744; doi:10.1038/s41598-019-54668-z)
Supplement: Supplementary file 1 — Suplementary Information [file 41598_2019_54668_MOESM1_ESM.pdf]

Supplementary Information

Anomalous Stranski-Krastanov growth of (111)-oriented quantum dots with tunable wetting layer thickness

Christopher F. Schuck[1,†], Simon K. Roy,[2] Trent Garrett,[2] Qing Yuan,[3] Ying Wang,[3] Carlos I. Cabrera,[4] Kevin A. Grossklaus,[5] Thomas E. Vandervelde,[5] Baolai Liang,[3] and Paul J. Simmonds[1,2,*]

[1] Micron School of Materials Science & Engineering, Boise State University, Boise, Idaho 83725, USA

[2] Department of Physics, Boise State University, Boise, Idaho 83725, USA

[3] College of Physics Science & Technology, Hebei University, Baoding 071002, P.R. China

[4] Center for Research in Sciences, Research Institute in Basic and Applied Sciences, Autonomous University of the State of Morelos, Av. Universidad 1001, 62209, Cuernavaca, Morelos, Mexico

[5] Department of Electrical and Computer Engineering, Tufts University, 161 College Avenue, Medford, Massachusetts 02155, USA

[†] Current address: Materials Growth Facility, University of Delaware, Newark, DE, 19716, USA

** Address correspondence to Paul J. Simmonds: paulsimmonds@boisestate.edu*

S1. Results and Discussion

S1.1 AFM: WL Growth Beyond the t_c

Using atomic force microscopy (AFM), we calculate the total volume of GaAs in the tensile-strained quantum dots (TSQDs) per unit area by multiplying average TSQD volume by areal density. To find the total volume of material in the wetting layer (WL) per unit area, we subtract the volume of material in the TSQDs from the amount of GaAs deposited in monolayers (ML), assuming a Ga sticking coefficient of unity at 535 ºC[1]. For example, 2.5 ML GaAs deposition corresponds to a volume of 423,613 nm^3/µm^2, the total volume in the TSQDs is 284 nm^3/µm^2, and therefore the WL volume is 423,329 nm^3/µm^2. 4.5 ML GaAs corresponds to a volume of 762,503 nm^3/µm^2, the total volume in the TSQDs is 4,335 nm^3/µm^2,

and therefore the WL volume is 758,167 $nm^3/\mu m^2$. This data suggests that at 2.5 ML and 4.5 ML GaAs deposition, only 0.07% and 0.57% of the GaAs incorporates into the TSQDs, respectively, consistent with an increase in WL volume.

S1.2 Confirmation of Photoluminescence Peak Assignments

Excitation-density-dependent photoluminescence (PL) of our TSQD samples allows us to rule out excited state emission as the origin of the primary peak. As we raise the excitation density from 0.3–3000 W/cm^2, Fig. S1 shows the resulting PL emission from the 4.5 ML GaAs TSQD sample.

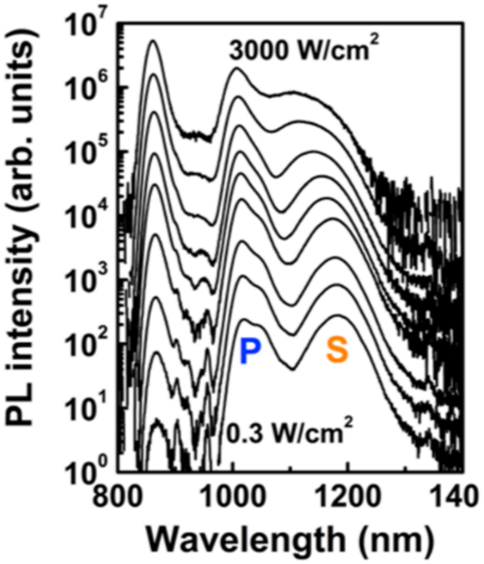

Figure S1. Excitation-density-dependent PL of a 4.5 ML GaAs TSQD sample at 7 K. (a) Change in PL spectra as the excitation intensity is increased from 0.3–3000 W/cm^2. Labels indicate the primary (P) and secondary (S) GaAs peaks.

Note that we chose to use the 4.5 ML sample to illustrate our findings in the interest of clarity, since it has the largest primary-secondary peak separation [Fig. 2]. These results are, however, representative of the other TSQD samples. For a sample without WL emission, at low pump powers we would expect to see only a single PL peak corresponding to emission from the TSQD ground states. Increasing the pump power would eventually lead to a saturation of the TSQD ground states, and then appearance of a second

peak at shorter wavelength corresponding to emission from the first excited state of the TSQDs. The fact that we see the shorter wavelength primary peak even for excitation densities as low as 0.3 W/cm^2, suggests that this is not emission from an excited state of the TSQDs.

S1.3 Cross-sectional transmission electron microscopy (XTEM)

We produced XTEM samples using a focused ion beam (FIB) lift-out method. We did XTEM imaging in a JEOL ARM 200F aberration-corrected microscope operated at an accelerating voltage of 200 kV. In all cases, we aligned the samples to a [110] zone-axis. In bright-field TEM we can directly see that the 4.5 ML GaAs TSQDs are dislocation-free despite the high tensile strain of 3.8% [Fig. S2].

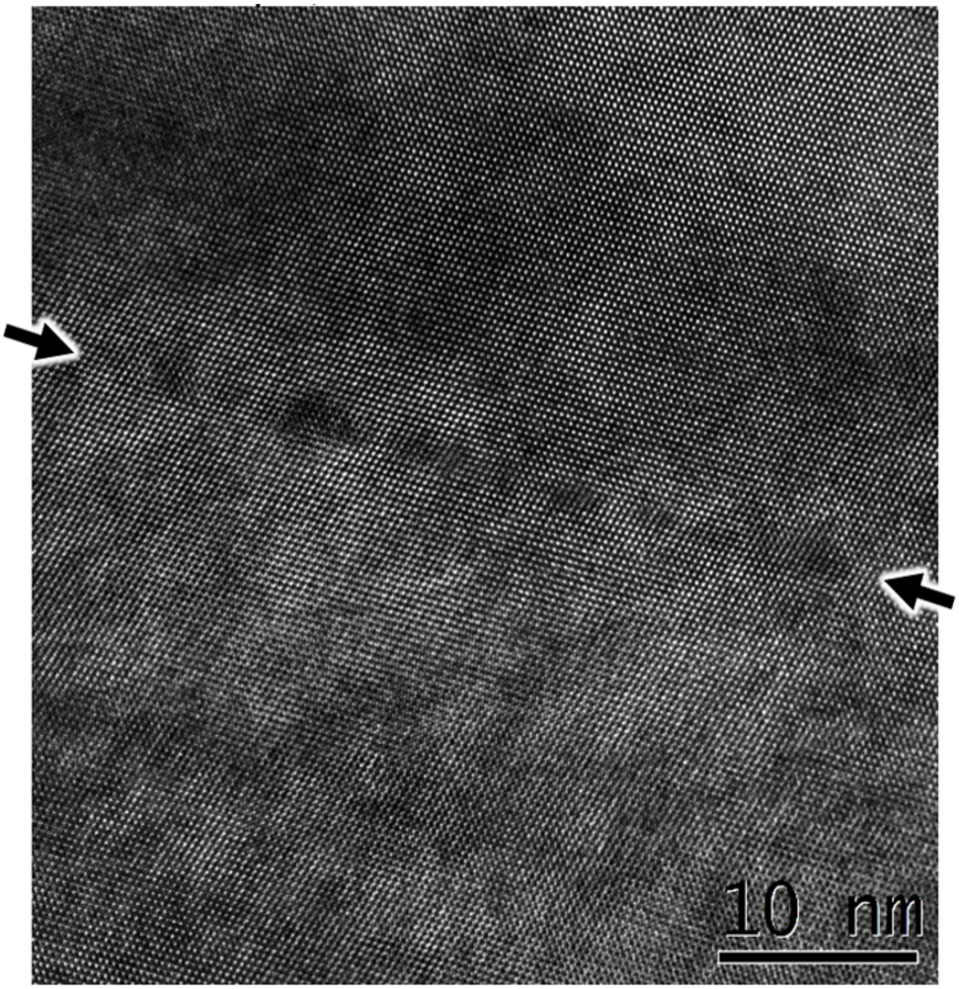

Figure S2. A [110] zone-axis high resolution bright-field TEM image of a 4.5ML sample showing TSQDs visible due to strain contrast. Arrows indicate the layer containing the WL and TSQDs. No crystalline defects are visible in the InAlAs barriers or GaAs TSQDs.

S1.4 TSQD & WL Computational Model

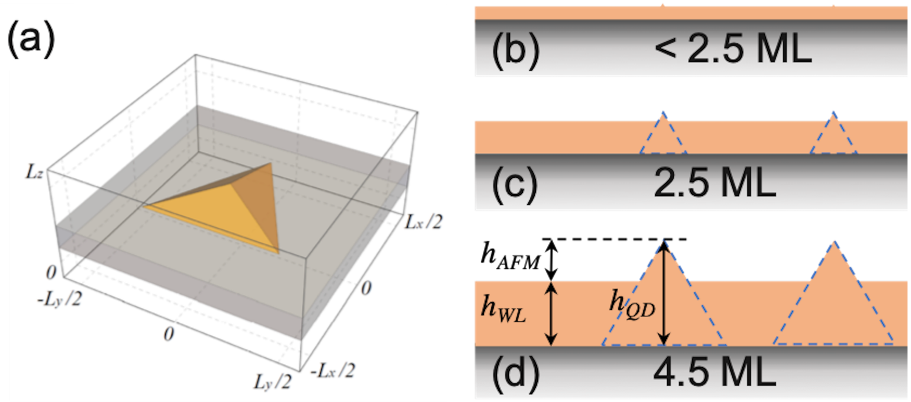

Figure S3. (a) Schematic example of the geometric TSQD configuration used in the model. (b)–(d) Diagrams illustrating the effective TSQD height, as adopted from Wang[2]. For the modified SK growth mode suggested by our experimental and computational results, both h_{AFM} and h_{WL} are variables. (b) Growth begins with the initial formation of a 2D WL. (c) At t_c = 2.5 ML, transitions to the self-assembly of 3D TSQDs. (d) Further GaAs deposition leads to larger TSQDs *and* a thicker WL.

S2. Experimental Methods

We grow GaAs/InAlAs TSQD samples on on-axis InP(111)A substrates using solid-source molecular beam epitaxy (MBE). We measure deposition amount in ML, and growth rates in ML/s from reflection high-energy electron diffraction (RHEED) intensity oscillations. We calibrate substrate temperature by comparing thermocouple and pyrometer readings against known changes in the surface reconstruction observed with RHEED. We calculate V/III beam equivalent pressure ratios using a beam flux monitor. All samples have the same structure [Fig. S4 (a)]: 40 nm $In_{0.53}Ga_{0.47}As$ smoothing buffer (172 nm/hr, 510 °C), 160 nm $In_{0.52}Al_{0.48}As$ bottom barrier, (169 nm/hr, 510 °C), 0 – 4.5 ML embedded GaAs TSQDs for optical analysis (0.075 ML/s, 535 °C, 75 As_4/Ga), 40 nm InAlAs top barrier (172 nm/hr: first 10 nm at

535 ºC, remaining 30 nm at 510 ºC), and 0 – 4.5 ML surface GaAs TSQDs for structural analysis (0.075 ML/s, 535 °C, 75 As_4/Ga).

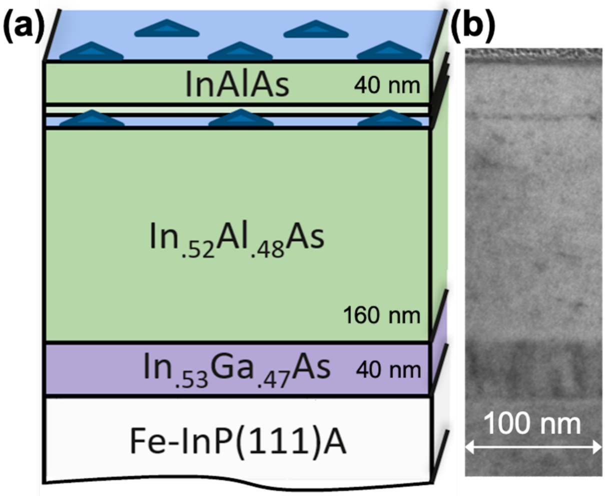

Figure S4. (a) A schematic of the sample heterostructure containing GaAs(111)A TSQDs (blue triangles). (b) XTEM image for comparison showing the expected sample structure.

We image the samples using cross-sectional transmission electron microscopy (XTEM), confirming that they are free from threading dislocations and have good crystal quality [Fig. S4 (b)]. RHEED shows a streaky 2×2 surface reconstruction during all bulk and TSQD growth[3–6].We confirm the InGaAs and InAlAs layers are lattice-matched to InP using x-ray diffraction. We use a valved arsenic cracker at 600 ºC to supply As_4, for consistency with previous studies of growth on (111)A surfaces[6–9]. The InAlAs barriers provide 3.8% tensile lattice-mismatch with the GaAs to drive TSQD self-assembly, while also offering carrier confinement within the TSQDs[10,11].

References

1. Kawaharazuka, A. & Horikoshi, Y. Behavior of Ga atoms deposited on GaAs (111)B and (111)A surfaces. *J. Cryst. Growth* **477**, 25–29 (2017).

2. Wang, L. *et al.* Self-assembled quantum dots with tunable thickness of the wetting layer: Role of vertical confinement on interlevel spacing. *Phys. Rev. B* **80**, 085309 (2009).

3. Fahy, M. R., Sato, K. & Joyce, B. A. Reflection high-energy electron diffraction intensity oscillations during the growth by molecular beam epitaxy of GaAs(111)AFahy. *Appl. Phys. Lett.* **64**, 190–192 (1994).

4. Chadi, D. J. Atomic and electronic structures of (111), (211), and (311) surfaces of GaAs. *J. Vac. Sci. Technol. B* **3**, 1167–1169 (1985).

5. Katnani, A. D. & Chadi, D. J. Photoemission and theoretical studies of GaAs(111) and (-1-1-1) surfaces: Vacancy models. *Phys. Rev. B* **31**, 2554–2556 (1985).

6. Schuck, C. F. *et al.* Self-assembly of (111)-oriented tensile-strained quantum dots by molecular beam epitaxy. *J. Vac. Sci. Technol. B* **36**, 031803 (2018).

7. Cho, A. Y. Growth of III–V semiconductors by molecular beam epitaxy and their properties. *Thin Solid Films* **100**, 291–317 (1983).

8. Simmonds, P. J. & Lee, M. L. Tensile-strained growth on low-index GaAs. *J. Appl. Phys.* **112**, 054313 (2012).

9. Yerino, C. D. *et al.* Strain-driven growth of GaAs(111) quantum dots with low fine structure splitting. *Appl. Phys. Lett.* **105**, 251901 (2014).

10. Yerino, C. D. *et al.* Tensile GaAs(111) quantum dashes with tunable luminescence below the bulk bandgap. *Appl. Phys. Lett.* **105**, 071912 (2014).

11. Simmonds, P. J. *et al.* Tuning Quantum Dot Luminescence Below the Bulk Band Gap Using Tensile Strain. *ACS Nano* **7**, 5017–5023 (2013).
